# AI and IoT in Sugar Beet Systems: A Review of Monitoring, VOC Sensing, and Post-Harvest Applications

**DOI:** 10.3390/s26134072

**Published:** 2026-06-26

**Authors:** Bakht Alam Khan, Sulaymon Eshkabilov

**Affiliations:** Department of Agricultural and Biosystems Engineering, North Dakota State University, Fargo, ND 58108, USA; bakht.khan@ndsu.edu

**Keywords:** sugar beet, post-harvest storage, sensors, electronic nose, volatile organic compounds, internet of things, machine learning, artificial intelligence

## Abstract

The global sugar industry is facing increasing challenges due to climate variability, sustainability requirements, and the need for improved operational efficiency. These pressures are driving the search for advanced technological solutions to enhance productivity and resource management. Artificial intelligence (AI) has already demonstrated significant potential across various agricultural sectors; however, a comprehensive evaluation of AI applications across the entire sugar industry value chain from crop cultivation to industrial processing and supply chain management remains limited. This review provides a detailed assessment of the current state of AI and internet of things (IoT) implementation in the sugar beet industry. It examines key applications, including precision agriculture for sugarcane and sugar beet cultivation, intelligent monitoring systems for early disease detection, and AI-driven decision support tools for resource optimization. In addition, the study explores the role of AI in sugar manufacturing processes, where machine learning and data-driven models are used to optimize milling operations, improve product quality control, and enable predictive maintenance of industrial equipment. AI technologies are also shown to enhance supply chain efficiency through improved demand forecasting, logistics optimization, and real-time data analytics. Monitoring volatile organic compounds (VOCs) is becoming increasingly important in sugar beet and sugarcane storage. Microbial activity during storage and fermentation can release VOCs such as ethanol, which act as early indicators of crop degradation and spoilage. Detecting these gases using modern gas sensors enables continuous monitoring of storage conditions and crop health. When sensor data is integrated with AI and IoT systems, it can be analyzed in real time to identify early signs of microbial activity, improve storage management, and optimize processing decisions. Such intelligent monitoring systems have the potential to reduce losses and enhance overall efficiency in the sugar production chain.

## 1. Introduction

The sugar industry plays a crucial role in the global agricultural economy [1], acting as a major supplier of one of the most widely consumed food commodities and contributing significantly to the economic development of many countries [2,3]. This industry includes both sugarcane and sugar beet production systems, each of which is currently facing a range of complex challenges in the modern agricultural landscape [4,5]. These challenges include the increasing need for sustainable production practices as well as the ongoing demand to improve operational efficiency throughout the supply chain [2]. For example, India has emerged as one of the most influential players in the global sugar market, becoming both the largest producer and consumer of sugar worldwide, which further highlights the global importance of the industry [6,7]. The magnitude of the global sugar market emphasizes its economic significance. In 2023, the market was valued at approximately USD 66.39 billion and is expected to grow at a compound annual growth rate (CAGR) of about 6.5% through 2030 [8]. At present, the sugar sector is operating within a rapidly changing and highly uncertain environment. One of the major challenges is the increasing variability in climate conditions, which can directly influence the availability, quality, and consistency of raw agricultural materials. These climate-related fluctuations often lead to variations in crop yield and ultimately affect overall sugar production levels [9]. In addition to environmental challenges, sugar producers are also facing growing pressure from regulatory bodies and society to adopt more environmentally responsible practices. This includes reducing energy consumption, lowering greenhouse gas emissions, and minimizing the overall carbon footprint of production processes [9]. Addressing these interconnected challenges requires a proactive and adaptive strategy that integrates modern technological innovations into traditional production systems. By leveraging advanced technologies such as artificial intelligence, data analytics, and smart automation, the sugar industry can better manage climate-related risks while simultaneously improving energy efficiency and operational performance across the entire production cycle [2].

Producing sugar from sugar beet is often considered more economically favorable than extracting sugar from sugarcane because sugar beet typically contains approximately 25% more sugar than sugarcane [10]. However, once harvested, the chemical composition of sugar beet begins to change during storage, which gradually reduces the amount of recoverable sugar. One of the main processes responsible for this change is the activity of sucrolytic enzymes, which break down sucrose into glucose and fructose. These hexose sugars are primarily consumed during the respiration processes of beet tissues, although a portion may accumulate within the cells [11]. In addition to temperature, storage duration plays a critical role in determining the technological quality of stored sugar beet [12]. This quality depends not only on the sucrose concentration but also on the presence of certain compounds that interfere with sugar crystallization during the refining process [13]. After harvesting, the formation of invert sugar results mainly from the hydrolysis of sucrose and metabolic respiration processes occurring within beet roots [14].

During the initial hours of storage, the concentration of invert sugar may decrease due to reduced invertase enzyme activity and the utilization of reducing sugars in metabolic reactions within the beet tissue [15]. Over time, however, the amount of invert sugar gradually increases at a rate influenced by storage conditions and the physiological health of the beet. Furthermore, different sugar beet cultivars vary in both the quantity of sugar accumulated during the growing season and their behavior during storage [16]. The concentration of invert sugar in sugar beet is widely recognized as an important indicator of technological quality, as it reflects the breakdown of sucrose and may contribute to the formation of undesirable organic acids during subsequent processing. Variations in climatic conditions have led to the development and cultivation of sugar beet hybrids that are better adapted to changing environmental conditions. Understanding the storage behavior of these hybrids and maintaining their quality is therefore essential for efficient sugar production, since the quality of raw material directly affects processing performance and final product yield. Previous studies have examined the storage behavior of sugar beet over periods of up to 60 days, which typically represents the maximum duration between harvest and industrial processing.

Post-harvest losses remain a critical global challenge with far-reaching implications for food security, economic development, and environmental sustainability [17,18,19]. A significant proportion of the food produced worldwide never reaches consumers due to losses that occur at various stages of the supply chain. According to the United Nations Food and Agriculture Organization (FAO), nearly one-third of all food produced for human consumption, approximately 1.3 billion tons, is either lost or wasted every year, representing an estimated economic loss of around USD 990 billion globally [20,21]. These losses reflect an inefficient utilization of agricultural resources such as land, water, labor, and energy, while simultaneously intensifying the global challenge of food insecurity faced by many countries [22]. As the global population continues to grow and the demand for food increases, reducing post-harvest losses has become an essential priority for improving the efficiency and sustainability of food systems.

Food losses can occur throughout the entire agricultural supply chain, beginning with primary agricultural production and continuing through harvesting, post-harvest handling, storage, processing, distribution, retail, and ultimately household consumption [23]. Studies indicate that approximately 54% of total food losses occur during the early or upstream stages of the supply chain, particularly during production, harvesting, and storage operations. The remaining 46% occur during downstream processes, including processing, transportation, retail distribution, and final consumption [24]. The impacts of these losses extend beyond economic costs, affecting food availability, market stability, and environmental sustainability [25]. For example, in sub-Saharan Africa, it is estimated that nearly 37% of post-harvest losses occur before agricultural products reach the processing stage [26]. These losses are primarily attributed to ineffective harvesting techniques, inadequate storage infrastructure, insufficient processing facilities, and limited access to modern agricultural technologies [27]. Improving post-harvest management practices could therefore lead to significant economic and social benefits. In Ethiopia alone, reducing post-harvest losses could potentially provide sufficient food for approximately 23 million people while saving the government an estimated USD 1.2 billion annually in food import costs [25].

Addressing the issue of post-harvest losses requires a comprehensive and integrated approach that considers technological, economic, cultural, and logistical factors within agricultural and food systems [28]. Traditional methods of loss reduction are often insufficient in addressing the complexity of modern food supply chains. As a result, increasing attention has been directed toward the use of advanced digital technologies that can improve monitoring, transparency, and decision-making across the supply chain. Emerging technologies such as blockchain and artificial intelligence (AI) have shown considerable potential for improving post-harvest management by enhancing traceability, optimizing logistics, and enabling data-driven decision-making processes [29,30]. Studies have suggested that the implementation of technological solutions in storage facilities alone could reduce post-harvest losses from as high as 50–60% to as little as 1–2% when appropriate monitoring and management systems are applied [31]. These findings highlight the transformative potential of technological innovations in minimizing food losses and improving supply chain efficiency.

Post-harvest losses also have significant consequences for global food security and economic resilience. In regions where agricultural productivity is already constrained by limited resources and infrastructure, such losses further reduce food availability and increase economic vulnerability. In sub-Saharan Africa, for instance, substantial portions of harvested crops are lost before reaching processing facilities, contributing to economic losses and worsening food insecurity for millions of people [25]. Additionally, post-harvest losses increase market prices and place additional pressure on agricultural production systems to compensate for wasted food. This inefficiency also results in environmental degradation, as valuable resources such as water, fertilizers, energy, and land are consumed to produce food that ultimately goes unused [32]. Consequently, reducing post-harvest losses is not only essential for improving food availability but also for promoting sustainable agricultural practices and reducing environmental impacts. Intelligent monitoring systems equipped with sensors can also continuously track environmental conditions such as temperature, humidity, and gas emissions within storage environments, allowing for early detection of spoilage and enabling timely intervention [33]. These capabilities support more efficient and data-driven decision-making processes that can significantly reduce post-harvest waste and improve operational performance across the food supply chain.

Recent technological advancements have further increased interest in how AI can be applied across economic sectors, including agriculture, to generate value and improve productivity [33,34]. AI encompasses a broad range of technologies and analytical tools, including predictive modeling, machine learning algorithms, image recognition systems, and intelligent monitoring platforms [35,36,37]. These technologies have already demonstrated considerable value in agricultural applications such as precision farming, crop monitoring, yield prediction, and resource management [33]. By integrating AI into post-harvest operations, agricultural stakeholders can improve the efficiency of storage, transportation, and processing systems while minimizing product deterioration and quality loss. Consequently, the strategic deployment of AI-based technologies within post-harvest management systems offers a powerful approach for addressing persistent challenges within the agricultural sector and strengthening global food security.

As the adoption of AI technologies in agriculture continues to expand, it becomes increasingly important to systematically evaluate how these technologies align with the specific tasks involved in post-harvest management [38,39]. While previous studies have explored the general potential of AI in agriculture, relatively few have examined how individual AI capabilities can effectively support specific post-harvest activities such as storage monitoring, sorting, grading, and logistics optimization [40]. To address this gap, the present study conducts a comprehensive review of existing literature using the Technology–Task Fit (TTF) framework. This framework provides a structured approach for evaluating how well technological capabilities match the requirements of specific operational tasks. By analyzing AI applications through this perspective, the study aims to identify opportunities for improving the design and implementation of AI-driven solutions that can effectively reduce post-harvest losses and enhance the efficiency and sustainability of agricultural supply chains. This review highlights volatile organic compounds (VOCs), their impact on crops, and the detection of VOCs using sensors and IoT techniques. It further explores how these approaches can be applied to sugar beets. Finally, the review discusses the use of artificial intelligence (AI) in sugar beet cultivation and post-harvest management. More research is required to understand how sugar beets can be effectively managed after harvest, especially during transport and storage. Although the sugar industry includes both sugarcane and sugar beet production, this review only focuses on the post-harvest management and storage monitoring of sugar beets. Sugarcane is mentioned only when comparisons are useful. In addition, the sensing and artificial intelligence methods discussed in this review may also be applied to the post-harvest monitoring of other agricultural products. Ensuring their health and implementing systems to quickly detect and report spoilage or fermentation are essential. Many existing reviews discuss artificial intelligence, IoT, and smart sensing in agriculture in a broad way. However, this review focuses specifically on sugar beet production and post-harvest storage monitoring. Its main contribution is that it brings together four connected areas: AI-based crop monitoring, IoT-based storage sensing, VOC and gas detection, and quality loss in stored sugar beet roots. This review gives special attention to ethanol, CO_2_, VOC patterns, temperature, humidity, and sensor-based data because these factors can help indicate microbial activity, fermentation, and early storage deterioration. This is important because sugar beet roots are usually stored in large piles, where temperature, moisture, aeration, and microbial activity can vary inside the pile. In such conditions, early spoilage may not be visible from the outside. Therefore, this review provides a focused overview of sensing technologies, AI methods, IoT systems, and practical challenges for developing smart sugar beet storage monitoring systems. The AI, IoT, and VOC sensing approaches for sugar beet monitoring are compared in Table 1 below.

## 2. Review Objectives

The objective of this review is to provide a comprehensive analysis of emerging technologies that can improve monitoring, quality assessment, and crop loss reduction in sugar beet production and post-harvest systems, and challenges in sugar beet storage monitoring. As storage losses and quality deterioration remain major challenges in the sugar beet industry, advanced monitoring and analytical technologies are becoming increasingly important for improving crop management and storage practices. In this context, the review focuses on sensing technologies and artificial intelligence that have been widely explored in agriculture and discusses their potential application in sugar beet systems. The review is organized into the following key themes:

### 2.1. Volatile Organic Compounds in Agricultural Products

The first objective is to provide an overview of volatile organic compounds (VOCs) and their role in biological and agricultural processes. VOCs are naturally produced during plant respiration, metabolic activity, and microbial interactions in crops. These compounds are widely studied as indicators of physiological changes, stress conditions, and post-harvest deterioration in agricultural products. This section discusses the origin and significance of VOCs in crops and explains how similar biochemical processes may also occur in sugar beet during storage, where microbial activity and respiration can generate volatile compounds associated with quality degradation.

### 2.2. Detection of VOCs in Crops Using Sensors and Electronic Nose Technologies

The second objective is to review sensing technologies used for detecting VOC emissions in agricultural products. Various gas sensing systems, including metal oxide semiconductor sensors, gas chromatography-based methods, and electronic nose (EN) technologies, have been widely used to monitor volatile compounds in fruits, vegetables, and other crops for early detection of spoilage or disease. Although many studies focus on other agricultural products, the principles of VOC detection can also be applied to sugar beet storage systems, where monitoring volatile emissions may help identify microbial activity and early stages of deterioration before visible symptoms appear.

### 2.3. Applications of Artificial Intelligence and IoT in Sugar Beet Production and Post-Harvest Management

The final objective of this review is to examine how artificial intelligence (AI) technologies have been applied in the sugar beet industry. AI-based methods are increasingly used to improve crop monitoring, disease detection, yield prediction, and precision agriculture practices during cultivation. In addition, AI is also being explored for post-harvest applications such as automated quality inspection, storage monitoring, and supply chain optimization. This section reviews current research on AI in sugar beet systems and highlights how intelligent data analysis can support improved decision-making and efficiency across different stages of the sugar beet value chain.

### 2.4. Literature Review Approach

The literature screening procedure was revised to provide a more systematic basis for the review. Relevant studies were identified from major scientific databases and publisher platforms, including ScienceDirect, Web of Science, MDPI, IEEE Xplore, DOAJ, PubMed/Google Scholar, and other relevant sources. The search strategy combined keywords associated with sugar beet storage, spoilage, volatile organic compounds, ethanol, CO_2_, gas sensing, electronic-nose systems, IoT, machine learning, artificial intelligence, and post-harvest monitoring. After the initial search, duplicate and clearly unrelated records were removed. The remaining studies were then screened based on their titles and abstracts, followed by full-text assessment for final selection. Studies included if they addressed one or more of the following areas: sugar beet storage deterioration, VOC- or gas-based spoilage detection, sensor and electronic-nose monitoring, IoT-supported agricultural monitoring, or AI/ML approaches applicable to crop and post-harvest decision support.

Studies were excluded if they were not related to sugar beet or post-harvest monitoring, lacked adequate methodological information, were not peer-reviewed, or focused only on broad agricultural applications without a clear connection to storage sensing, spoilage detection, or AI/IoT-based monitoring. This revised screening process helped ensure that the selected literature was directly relevant to the scope of the review and supported a more transparent selection of sources.

**Figure 1:**
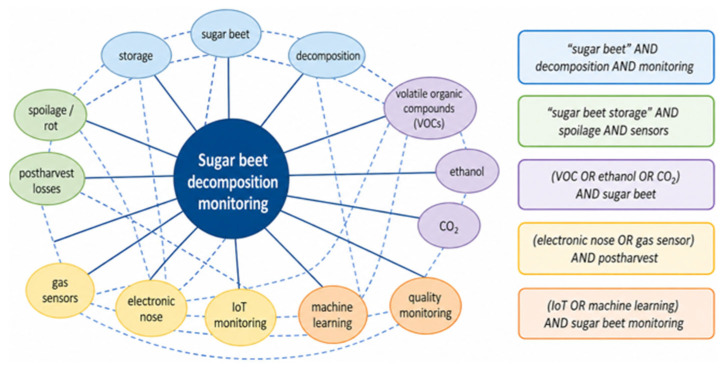
Keyword combination strategy used for the literature search on sugar beet decomposition monitoring.

Technology–Task Fit (TTF) as shown in Figure 1 was adopted as an organizing framework to assess the suitability of the reviewed technologies for the specific monitoring tasks required in sugar beet production and storage. Solid lines link the core concept to individual keywords, and dashed lines show the interconnections between those keywords. In this review, the key tasks were categorized as disease and stress detection during cultivation, storage environment monitoring, VOC/gas-based spoilage detection, real-time data transmission, and AI-supported prediction or classification. Each study was therefore interpreted according to the extent to which the applied technology addressed the intended monitoring function.

**Figure 2:**
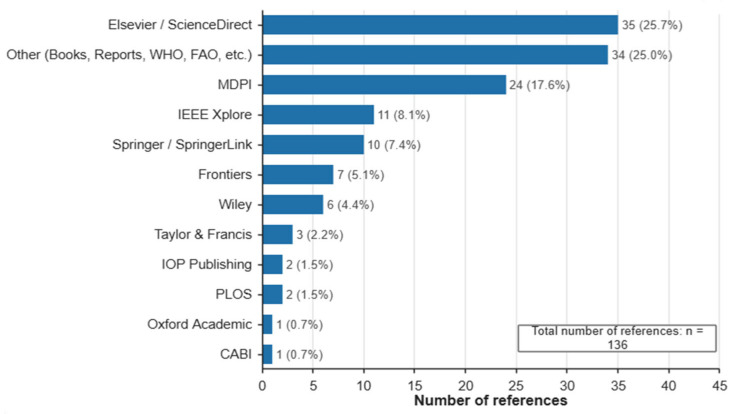
Database-wise distribution of research papers collected for review.

For example, computer vision methods, UAV imaging, and remote sensing approaches were primarily assessed in relation to field monitoring, canopy assessment, disease recognition, and plant-level stress detection. In contrast, VOC sensors, CO_2_ sensors, gas sensing arrays, and electronic nose systems were evaluated based on their relevance to detecting biochemical changes and spoilage indicators during storage. Similarly, IoT-based systems were considered mainly for their ability to support continuous sensing, data acquisition, wireless communication, and real-time monitoring, whereas ML and DL models were evaluated for their roles in classification, prediction, anomaly detection, and decision-support applications. The distribution of selected papers, categorized by source journal, is detailed in Figure 2.

## 3. Volatile Organic Compounds

Volatile organic compounds (VOCs) comprise a broad class of organic chemicals present in many products that readily evaporate into the atmosphere under ambient conditions. Due to their high volatility, mobility, and relative persistence, these compounds can be transported over long distances in the environment [47]. They are organic compounds that include hydrocarbons, alcohols, aldehydes, and organic acids. VOCs originate from both natural (biological) and human-made (anthropogenic) sources and can also be categorized based on their level of volatility [48]. Biogenic volatile organic compounds (BVOCs), mainly terpenoids like isoprene, alcohols, and carbonyls, are emitted by plants, animals, or microorganisms as byproducts of metabolism or decomposition, often for biological communication [49]. Examples include plants attracting pollinators, defending against herbivores, or animals signaling territory to deter competitors. BVOCs constitute the largest portion of atmospheric VOCs, where they participate in various atmospheric processes, primarily photochemical reactions. Their interactions with atmospheric radicals are particularly well studied [50].

### Volatile Organic Compound Sources

The time span of these organic compounds present in the atmosphere can last from hours to months. Anthropogenic VOCs originate primarily from fossil fuels, particularly due to incomplete combustion in vehicles, and from numerous human-made products and activities, including paints, adhesives, cosmetics, coatings, plastics, furniture, aerosols, perfumes, pesticides, flame retardants, cleaning agents, industrial processes, tobacco smoke, cooking, and many other sources [51]. Many VOCs can adversely affect the environment and pose significant health risks [52]. The severity of these risks varies, but while most VOCs are not immediately harmful, prolonged exposure can lead to toxic, carcinogenic, mutagenic, genotoxic, or teratogenic effects in humans [53]. Indoor air often contains higher VOC concentrations than outdoor air, making it a primary focus of research and regulation [54]. Environmentally, VOCs contribute to photochemical smog by reacting with nitrogen oxides (NOx) to produce ozone [55] and can also generate secondary particulate matter [56]. These processes can reduce crop yields, alter cloud formation and precipitation, damage crops and infrastructure, and contribute to global warming [57].

Analyzing VOCs is essential for promoting sustainability and improving quality of life. VOC analysis enables monitoring of air quality, identification of pollution sources, and support for strategies aimed at reducing environmental exposure. Assessing both indoor and outdoor air can help minimize human exposure to harmful VOCs. Characterizing VOCs also aids in identifying and limiting the use of hazardous chemicals in consumer products, encouraging more sustainable and eco-friendly alternatives. Additionally, monitoring VOCs in food products ensures safety for consumption and compliance with regulatory standards. Sampling VOCs presents several challenges. Because VOCs are often diluted in air, preconcentration is frequently required [58], which can be achieved using sorption tubes or cryogenic traps [59]. However, VOCs may react with surfaces, with each other, or be affected by water accumulation, potentially compromising samples during collection, transport, or storage. Trace VOCs can also be captured using solid phase microextraction (SPME) [60]. To reduce these issues, samples can be collected in gas bags, syringes, or glass or steel containers without preconcentration. In some cases, freezing the samples is necessary to preserve them, while in other instances, some degree of sample degradation is inevitable [61,62,63,64,65]. The World Health Organization categorizes volatile organic compounds into subcategories such as VVOCs, VOCs and SVOCs considering their boiling point ranges (see Table 2) [66].

Volatile organic compounds (VOCs) are organic chemicals such as hydrocarbons, alcohols, aldehydes, ketones, and organic acids that can easily evaporate under normal environmental conditions. Volatile organic compounds are key precursors in ozone formation and can significantly deteriorate air quality [67]. In agricultural and postharvest systems, VOCs are important because they can be released during crop respiration, microbial activity, tissue injury, fermentation, and decomposition. Changes in VOC type and concentration can therefore provide useful signals about crop freshness, spoilage progression, and storage quality. As a result, VOC monitoring offers a non-destructive approach for detecting early biological deterioration in stored agricultural products.

In storage environments, VOC patterns may appear before visible spoilage symptoms, making them valuable for early warning systems. This is especially useful for crops stored in bulk, where internal quality changes are difficult to observe manually. When VOC sensing is combined with environmental measurements such as temperature, humidity, and CO_2_, it can provide a more complete understanding of storage conditions. Such monitoring can support timely decision-making, reduce postharvest losses, and improve the management of stored crops. Therefore, VOC-based detection has strong potential for developing smart postharvest monitoring systems, particularly when integrated with IoT and data-driven analysis tools. In stored sugar beet roots, VOC release is strongly associated with biochemical changes and microbial deterioration. Even after harvest, the roots remain metabolically active, continuing to respire and use stored sucrose, which leads to the production of CO_2_ and heat [68]. When sugar beet roots are damaged, stored under unsuitable temperatures, or exposed to poor ventilation and excessive moisture, microbial growth may become more active. These microorganisms can metabolize accessible sugars and injured tissues, promoting fermentation and the release of volatile compounds, including ethanol and organic acids [69]. Therefore, shifts in VOC profiles should not be interpreted as random gas release. Instead, they indicate the biological processes occurring within stored sugar beet roots, including respiration, sucrose breakdown, tissue damage, and microbial decomposition [70]. While a wide variety of volatile compounds are emitted from agricultural products, recent analytical advancements have clarified which specific VOCs serve as the most reliable biomarkers for sugar beet deterioration during post-harvest storage. Among these, ethanol and ethyl acetate have been identified as the most consistent and measurable indicators of early-stage storage rot [71].

**Figure 3:**
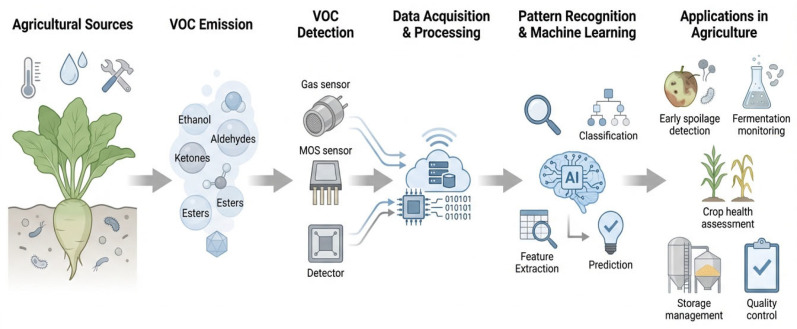
VOCs from agricultural sources detection and monitoring (figure created using Figure Labs AI-assistance).

Figure 3 illustrates the overall workflow for monitoring in agricultural environments using sensor-based and artificial intelligence (AI)-driven systems. The process begins with agricultural sources, including crops, soil, and environmental stress factors such as temperature and humidity, which influence plant metabolism and microbial activity. These biological and environmental interactions result in the emission of volatile compounds, including ethanol, aldehydes, ketones, and esters, which serve as indicators of physiological changes and degradation processes. These emissions are captured and measured using a range of sensing technologies, such as gas sensors and metal oxide semiconductor (MOS) sensors, which respond to different chemical compounds. The sensor outputs are then transmitted to data acquisition systems, where analog signals are converted into digital form and stored or processed in cloud-based or embedded platforms.

Subsequently, advanced data processing techniques are applied, including feature extraction and pattern recognition algorithms. Machine learning models analyze these patterns to identify trends, classify conditions, and generate predictive insights related to crop status. The final stage of the framework highlights key agricultural applications, including early detection of spoilage, monitoring of fermentation processes, crop health assessment, storage optimization, and quality control. This integrated approach enables real-time monitoring and supports informed decision-making, ultimately improving efficiency, reducing post-harvest losses, and enhancing sustainability in agricultural systems.

## 4. Detection of Volatile Organic Compounds

A variety of gas sensing technologies are available for detecting volatile compounds. Due to their limited selectivity, these sensors are primarily applied to estimate overall VOC levels rather than identifying individual chemical species. In most cases, they are capable of indicating the presence and approximate concentration of volatile substances in the atmosphere but cannot differentiate between specific compounds. Common sensor technologies in this group include photoionization detectors (PIDs), electrochemical sensors (ECS), and metal oxide sensors (MOSs).

**Figure 4:**
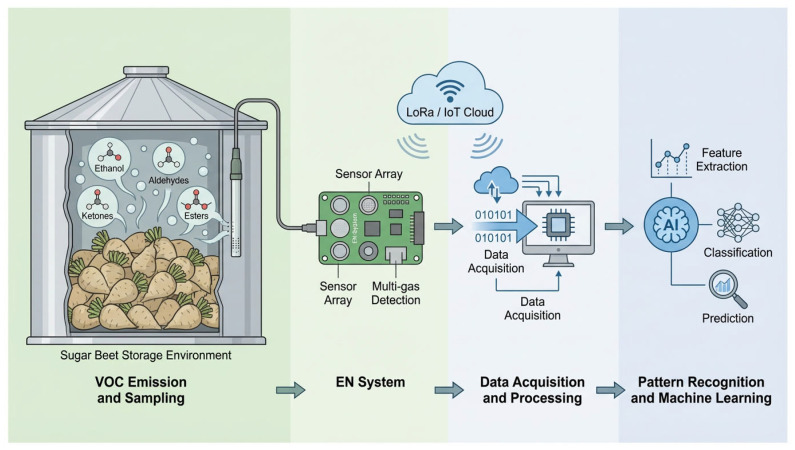
Volatile Organic Compounds from agricultural sources detection and monitoring (figure created using FigureLabs AI-assistance).

As illustrated in Figure 4, an integrated IoT-based electronic nose system is used to monitor VOC emissions from sugar beet storage environments as well as other crops or fruits. The system captures volatile compounds such as ethanol, aldehydes, ketones, and esters using a multi-sensor array. The collected data are transmitted through a LoRa-enabled cloud platform for real-time acquisition and processing. Advanced machine learning techniques are then applied for feature extraction, classification, and prediction of crop conditions. This approach enables early detection of spoilage and supports efficient storage management of sugar beets. The electronic nose (EN) system is designed to replicate the functioning of the mammalian olfactory system by using a sensor array capable of detecting changes in aroma and odor generated by volatile metabolites. This sensing mechanism allows the EN to recognize variations in volatile compounds released from biological samples, making it a useful analytical tool for monitoring chemical and biological processes [72]. Typically, an EN device consists of a collection of chemical sensors that respond selectively to different volatile compounds emitted by a sample. An example of such a system is the PEN-3 electronic nose, which incorporates ten distinct sensors (S1–S10), each designed to detect specific groups of gases. These sensors respond to compounds such as aromatic gases, nitrogen oxides, ammonia, hydrogen, methane, sulfur-containing gases, alcohols, and alkanes [73]. Different EN models are constructed with varying sensor configurations depending on the target compounds and the intended analytical application. In most electronic nose experiments, samples are placed inside sealed containers or vials to allow volatile compounds to accumulate in the headspace above the sample. The headspace gas, which may be analyzed with or without incubation, is then introduced into the sensor chamber of the electronic nose. When volatile molecules interact with the sensor surfaces, they cause changes in the physical or chemical properties of the sensing materials. These changes may include variations in electrical resistance, conductivity, optical properties, or mass accumulation depending on the sensor type. The resulting changes are converted into electrical signals that can be measured and analyzed [74,75].

The signals produced by each sensor within the array are combined to generate multidimensional datasets that represent the odor profile or volatile fingerprint of the analyzed sample. Because the EN system relies on the collective response of multiple sensors, it can detect subtle variations in volatile metabolite emissions. This ability enables early identification of biochemical processes such as microbial activity, crop maturation, or product degradation [76].

Several studies have demonstrated that electronic nose systems can effectively detect and differentiate changes in volatile compounds associated with crop development, disease progression, quality deterioration, shelf-life evaluation, and sensory attributes of agricultural products [77,78,79]. As a result, EN technology has been increasingly applied in agricultural and food monitoring systems for real-time assessment of crop conditions and product quality. Compared with conventional analytical techniques such as polymerase chain reaction (PCR), enzyme-linked immunosorbent assay (ELISA), and gas chromatography–mass spectrometry (GC-MS), electronic nose systems offer several advantages. These devices are generally simpler to operate, portable, relatively inexpensive, and capable of providing rapid results without requiring complex laboratory procedures [80]. Additionally, EN systems are able to analyze complex mixtures of volatile compounds by detecting characteristic odor patterns through the combined responses of multiple gas sensors [77,81]. Accurate quantification of VOCs in sugar beet matrices requires careful calibration due to matrix complexity and potential interactions between analytes and sample components. Variations in storage conditions, sample preparation, and matrix effects can significantly influence VOC detection and lead to discrepancies between calibration standards and real samples. Therefore, calibration strategies must be designed to closely replicate actual sample conditions to ensure reliable analytical performance. In this context, static headspace gas chromatography–mass spectrometry (HS-GC-MS) has been widely applied for VOC profiling, enabling systematic evaluation of calibration approaches for improved detection accuracy and reproducibility Biomarkers of Rotted Sugar Beet: A Low-Temperature VOC Analysis Framework [71].

The effectiveness of an electronic nose system largely depends on the configuration of the sensor array. Sensors with different selectivity characteristics are combined to detect a wide range of volatile compounds and produce unique signal patterns. These patterns can then be interpreted using pattern recognition algorithms to classify and identify volatile fingerprints associated with specific biological conditions [77]. In many cases, datasets from known samples are used to train machine learning or statistical models, improving the accuracy of detection and classification. Several types of sensors are commonly used in electronic nose systems. These include metal oxide semiconductor (MOS) sensors, metal oxide semiconductor field-effect transistor (MOSFET) sensors, piezoelectric sensors, quartz crystal microbalance (QCM) sensors, electrochemical sensors, and surface acoustic wave (SAW) sensors [82,83,84,85,86,87]. Each of these sensing technologies operates through different mechanisms and offers specific advantages depending on the application.

Among these technologies, metal oxide semiconductor sensors are widely used in post-harvest monitoring systems due to their relatively low cost, durability, and ability to detect a broad range of gases. MOS sensors operate based on changes in electrical conductivity that occur when gas molecules interact with the surface of a heated metal oxide layer. This interaction modifies the concentration of free electrons in the sensing material, leading to a measurable change in electrical resistance that can be correlated with the concentration of the target gas [82]. Although MOS sensors offer several advantages, they typically require relatively high operating temperatures ranging from approximately 300 °C to 500 °C. This requirement increases energy consumption compared with other sensing technologies [88]. Nevertheless, their reliability and sensitivity make them one of the most widely used sensors in electronic nose systems.

Another commonly used sensing technology is the MOSFET sensor, which detects gases by measuring changes in the threshold voltage of a field-effect transistor. When certain gases interact with the catalytic gate material, they alter the work function of the metal and oxide layers, resulting in a measurable change in the electrical output signal [83]. One of the advantages of MOSFET sensors is their high reproducibility due to fabrication using established microelectronic manufacturing techniques. Optical sensors represent another class of sensing technologies used in electronic nose systems. These sensors detect gases through changes in optical properties that occur when volatile molecules interact with specific sensing materials. Techniques such as absorption spectroscopy, fluorescence analysis, and surface plasmon resonance are commonly employed for gas detection [89]. For example, in absorption spectroscopy, the interaction between gas molecules and the sensing material can cause shifts in the absorption spectrum, allowing identification of specific gases. Similarly, fluorescence-based sensors detect changes in emitted light when the sensor material interacts with target compounds.

Optical sensors provide high sensitivity and selectivity, particularly for detecting low concentrations of gases. However, they are generally more expensive and may have limited operational lifetimes due to factors such as light source degradation or contamination of sensing materials [89]. Piezoelectric sensors, including quartz crystal microbalance (QCM) and surface acoustic wave (SAW) devices, are also widely used for detecting volatile compounds. These sensors operate based on changes in resonance frequency caused by the adsorption of gas molecules on the sensor surface. QCM sensors measure frequency changes in vibrating quartz crystals, while SAW sensors detect variations in acoustic wave propagation along the sensor surface [85,87]. These technologies offer high sensitivity and rapid response times, allowing detection of very small amounts of volatile compounds. Recent studies have emphasized the importance of sensor-based monitoring for understanding sugar beet decomposition dynamics during post-harvest storage. Decomposition is primarily driven by increasing microbial activity, which leads to the release of gases such as CO_2_ and ethanol, along with other volatile compounds that serve as early indicators of spoilage. Sensor systems, including gas sensors and electronic nose (E-nose) configurations, have been explored to capture these changes in real time. These approaches enable continuous monitoring of degradation processes and provide valuable insights into the progression of biochemical changes in stored sugar beets, supporting early detection and improved storage management Sensors for Sugar Beet Decomposition Dynamics Monitoring [90].

The choice of sensor type plays a crucial role in determining the performance of an electronic nose system. Each sensor responds differently to specific chemical compounds, generating characteristic signal patterns that can be analyzed using pattern recognition algorithms. These algorithms interpret sensor responses and convert them into meaningful information regarding the composition and concentration of volatile compounds present in the sample. The analysis of volatile metabolite profiles using electronic nose systems generally involves three major stages: (1) odor sampling, (2) detection of volatile compounds through sensor array analysis, and (3) data acquisition and pattern recognition. These steps enable the system to generate volatile fingerprints that can be used for classification, monitoring, and early detection of biological changes in agricultural products, as illustrated in Figure 4. The continued development of electronic nose technologies is therefore essential for improving crop monitoring, food safety, and quality assurance in modern agricultural systems. UV-driven activation of metal oxide sensors offers a promising route for detecting VOCs under room-temperature conditions. In one study, an SnO_2_/TiO_2_-based flexible sensor demonstrated a clear selective response to acetic acid vapor when exposed to UV light, suggesting that light-assisted sensing can enhance VOC detection performance without relying on high-temperature operation [91]. Visible-light-active photocatalysts, including Ni-modified gC_3_N_4_, have been reported as effective materials for degrading VOC pollutants. The improved performance of Ni-gC_3_N_4_ compared with undoped gC_3_N_4_ is mainly linked to stronger solar-light absorption, better charge-carrier separation, and enhanced photocatalytic stability during repeated use [92]. In sustainable agriculture, VOC emissions from plants and stored agricultural products can provide valuable information about biotic and abiotic stress responses. Detecting these compounds can therefore support continuous monitoring, early warning of potential deterioration, and improved risk management. The integration of nanoarchitecture sensing materials with machine learning-based pattern recognition can enhance the analysis of complex VOC responses and enable more reliable decision-making in crop and post-harvest monitoring applications [93]. One study indicates that CNT–metal oxide hybrid sensors may be useful for developing more sensitive and stable VOC monitoring systems for agricultural and post-harvest applications [94]. Type of sensors used to detect the volatile organic compounds along with their advantages can be seen in Table 3.

In addition, combining electronic nose systems with predictive analytics can support decision-making processes by forecasting potential quality degradation. As a result, these systems hold great potential for developing smart, automated agricultural monitoring solutions that ensure efficiency, sustainability, and rapid response to emerging issues. By combining sensor responses with data-driven algorithms, electronic nose systems can classify freshness levels, detect abnormal gas patterns, and support timely decision-making. Such systems can also reduce dependence on manual inspection, which is often time-consuming and subjective. In storage environments, continuous monitoring of VOCs, ethanol, CO_2_, temperature, and humidity can provide an early warning before visible spoilage occurs. Therefore, IoT- and AI-assisted sensing platforms offer a promising pathway for improving the safety, efficiency, and sustainability of postharvest crop storage.

**Figure 5:**
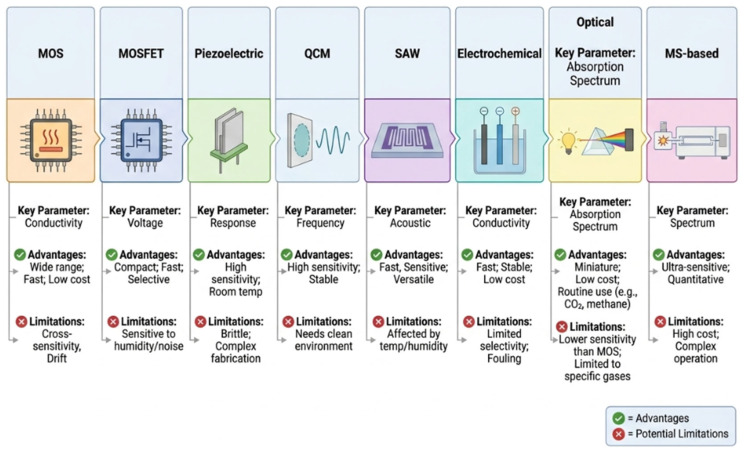
Comparison of major VOC and gas sensing technologies for postharvest monitoring.

Figure 5 summarizes the main sensing technologies commonly used for VOC and gas detection in agricultural and postharvest systems. MOS and electrochemical sensors are attractive because they are low-cost, fast, and suitable for portable monitoring, although they may suffer from cross-sensitivity and limited selectivity. MOSFET sensors provide compact and rapid detection, but their response can be affected by humidity and noise. Piezoelectric, QCM, and SAW sensors offer high sensitivity and can detect small changes in mass, frequency, or acoustic response caused by gas adsorption. However, these sensors often require stable operating conditions and may be affected by temperature, humidity, or fabrication complexity. Optical and MS-based methods provide highly sensitive and quantitative detection, making them useful for accurate VOC identification. Their main drawbacks are high cost, bulky instrumentation, and complex operation. Overall, the figure highlights the trade-off between affordability, sensitivity, selectivity, and practical field deployment for sugar beet storage monitoring. Electronic nose (e-nose) systems have emerged as rapid, cost-effective, and non-destructive tools for analyzing volatile organic compounds (VOCs) in food products [97].

## 5. AI and IoT in Sugar Beet Crop Production Systems

### 5.1. AI in Sugar Beet Cultivation

Artificial intelligence (AI) is increasingly being used to improve agricultural productivity, resource management, and crop monitoring. In sugar beet cultivation, AI technologies are applied in areas such as disease detection, crop health monitoring, yield prediction, and precision agriculture. Traditional farming methods often rely on manual observations and uniform management practices, which can lead to inefficient use of resources like water, fertilizers, and pesticides. In contrast, AI-based systems analyze large amounts of data from sensors, satellite imagery, drones, and field observations to support data-driven decision-making. Machine learning and computer vision techniques can identify patterns related to crop health and environmental conditions, allowing farmers to detect problems earlier and manage crops more efficiently. Furthermore, AI models can integrate temporal and spatial data to provide more accurate predictions of crop growth and yield under varying environmental conditions. These systems also enable site-specific management practices, where inputs are applied only where needed, improving efficiency and reducing environmental impact. In addition, AI-driven decision support tools can assist farmers in optimizing irrigation scheduling, nutrient management, and pest control strategies. The integration of AI with real-time monitoring systems further enhances responsiveness to changing field conditions, enabling proactive rather than reactive farm management. As a result, AI contributes to more sustainable, efficient, and resilient sugar beet production systems.

In sugar beet production, AI has been applied to tasks such as automated disease detection, plant identification using aerial imagery, and prediction of yield and crop quality. Deep learning models, particularly convolutional neural networks (CNN), have shown strong performance in identifying plant diseases and stress conditions from images of sugar beet leaves. In addition, machine learning (ML) models that integrate remote sensing data and environmental variables have been used to predict crop growth and yield more accurately. These developments highlight the potential of AI technologies to support precision agriculture and improve the efficiency of sugar beet cultivation systems.

The studies summarized in Table 4 demonstrate the growing role of AI in improving different aspects of sugar beet cultivation. One of the most widely explored applications of AI in this field is disease detection using deep learning models. Convolutional neural networks (CNN) and other deep learning architectures have shown strong performance in analyzing plant images to identify disease symptoms at early stages. These models can process large datasets of leaf images and automatically detect disease patterns that may not be easily visible through manual inspection. As reported in previous research, deep learning approaches have enabled accurate detection of sugar beet leaf diseases using image analysis techniques, improving the efficiency and reliability of crop monitoring systems [98].

In addition to image-based disease detection, hyperspectral imaging combined with machine learning (ML) algorithms has also demonstrated significant potential for early disease identification. Hyperspectral sensors capture detailed spectral information from plant tissues, which can reveal subtle biochemical changes associated with plant stress or infection. ML models applied to these spectral datasets are capable of distinguishing between healthy and infected plants with high accuracy. Such approaches allow early detection of plant diseases before visible symptoms appear, which can help farmers take timely management actions and reduce crop losses [99]. ML-based crop monitoring systems using unmanned aerial vehicles (UAVs) and computer vision technologies have further expanded the possibilities for large-scale agricultural monitoring. UAV platforms equipped with high-resolution cameras can capture aerial images of sugar beet fields, which are then analyzed using computer vision algorithms to evaluate plant health and detect stress conditions. These systems allow farmers to monitor large cultivation areas efficiently and identify problem zones that require targeted intervention. Research has shown that UAV imagery combined with ML models can effectively detect variations in crop health and environmental stress across sugar beet fields [100].

ML techniques have also been widely used for predicting crop yield and quality in sugar beet production. Artificial neural networks (ANNs) and other ML models can analyze complex relationships between environmental variables, soil properties, and crop growth parameters. By integrating historical agricultural data with real-time environmental information, these models can provide reliable predictions of root yield and sugar content. Such predictive tools help farmers and agricultural planners optimize management strategies and improve production efficiency [101]. Another important application of ML in sugar beet cultivation involves the use of deep learning models combined with remote sensing technologies for automated plant detection and field monitoring. Satellite imagery and remote sensing data provide valuable information about crop development and spatial variability within agricultural fields. ML-based image analysis techniques can automatically identify sugar beet plants and generate detailed maps of crop distribution across large agricultural areas. These automated monitoring systems support precision agriculture practices and enable more efficient management of sugar beet cultivation [102].

### 5.2. AI Applications in Post-Harvest Sugar Beet Processes

Artificial intelligence (AI) is increasingly being utilized to enhance post-harvest management and quality monitoring in sugar beet production. Following harvesting, sugar beet roots are highly vulnerable to mechanical injuries, microbial contamination, and biochemical degradation, all of which can significantly reduce sucrose concentration and processing efficiency during storage and transportation. Conventional inspection techniques largely depend on manual evaluation, which is labor-intensive and often inadequate for large-scale operations. In contrast, AI-driven solutions, including computer vision systems, machine learning models, and smart monitoring platforms offer advanced capabilities for automated quality assessment and storage optimization. These technologies facilitate accurate detection of damaged or diseased roots, classification of beet quality grades, continuous monitoring of storage environments, and improved logistics management.

Moreover, the integration of real-time sensor data with AI algorithms enables predictive analytics for early detection of spoilage and fermentation processes. This allows stakeholders to take timely corrective actions, minimizing losses and preserving product quality. Advanced AI models can also support decision-making by forecasting storage outcomes under varying environmental conditions. Additionally, the combination of IoT-enabled sensing systems with AI enhances traceability and transparency throughout the post-harvest supply chain. As a result, AI not only improves operational efficiency but also contributes to sustainable practices by reducing waste and optimizing resource utilization in sugar beet processing systems. When applying AI technologies in this context, it is important to distinguish between traditional ML methods and deep learning (DL) approaches. Conventional ML techniques, such as SVM and RF, are relatively lightweight and can efficiently handle structured IoT sensor data, making them well suited for deployment on low-power edge devices. On the other hand, DL models, including CNN and ANN with multiple layers, are designed to automatically learn relevant features from complex and unstructured inputs such as high-resolution images used for defect detection. DL models require high computing power, stable network access, and large labeled datasets. Therefore, they are generally more appropriate for cloud-based processing rather than direct use in limited-resource storage environments or real-time data analytics where data processing time is constrained.

### 5.3. Practical Limitations of AI- and IoT-Based Monitoring in Sugar Beet Storage Facilities

Practical deployment of AI- and IoT-based monitoring systems in sugar beet storage facilities is still associated with several challenges. First, sugar beet storage piles are large and highly heterogeneous; therefore, temperature, humidity, CO_2_, oxygen, and VOC levels may differ considerably between the surface, middle, and core regions of the pile [103]. As a result, data from only a few sensors may not accurately represent the overall storage environment. Second, harsh storage conditions, including high humidity, condensation, dust, soil particles, and microbial aerosols, can affect sensor stability, contribute to signal drift, and reduce long-term reliability [104]. Third, VOC sensors may suffer from cross-sensitivity because multiple gases, such as ethanol, acetaldehyde, acetic acid, ethyl acetate, CO_2_, and water vapor, can be released simultaneously during beet deterioration [105]. Fourth, wireless communication may be unstable inside dense beet piles or storage buildings, where signal attenuation, metal structures, and long distances from gateways can reduce data transmission quality [106]. Finally, AI models developed using laboratory or small-scale experimental data may not perform reliably in commercial storage piles unless they are recalibrated and validated under real storage conditions [107]. The deployment of IoT-enabled agricultural ground robots has emerged as a transformative approach for specialty and root crops, offering autonomous capabilities that significantly reduce labor inputs and improve precision sensing tasks [108].

**Figure 6:**
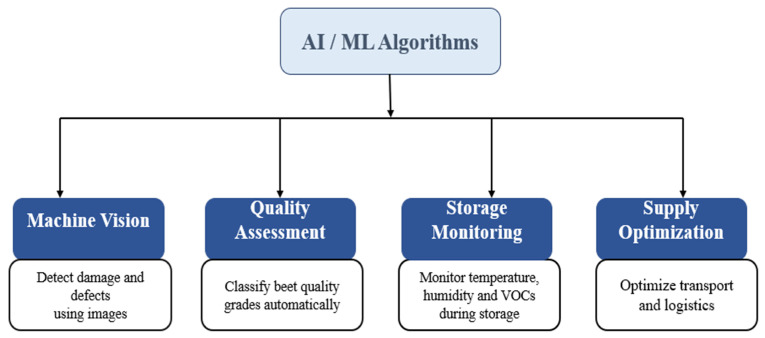
AI/ML used in post-harvest sugar beet processing.

Figure 6 presents a conceptual overview of how AI and ML techniques can be applied to improve post-harvest management of sugar beet. At the core of the framework are AI/ML algorithms that process information collected from imaging systems, sensors, and operational data to support different decision-making tasks. One important application is machine vision, where image-based analysis is used to identify physical damage, defects, or contamination on harvested sugar beet roots (Table 4). Automated quality assessment systems can also classify beet quality grades based on visual characteristics, enabling faster and more objective sorting during post-harvest handling. In addition, AI can support storage monitoring by analyzing data from environmental sensors to track temperature, humidity, and volatile compounds that may indicate deterioration during storage. AI-driven analytics can assist in supply chain optimization by improving transportation planning, storage scheduling, and logistics management. Together, these applications demonstrate how AI technologies can enhance efficiency, improve quality monitoring, and reduce losses in post-harvest sugar beet management. AI can support storage monitoring by analyzing data from environmental sensors that measure parameters such as temperature, humidity, and volatile organic compounds (VOCs), which may indicate biological activity or deterioration during storage. The applications shown in Figure 6 demonstrate how AI technologies can contribute to improved monitoring, quality management, and operational efficiency in post-harvest sugar beet systems. Moreover, the integration of AI with IoT-based monitoring platforms can enable continuous tracking of storage conditions and provide early warnings when abnormal patterns are detected. This can help operators take timely corrective actions, such as adjusting ventilation, temperature, or storage duration before severe quality losses occur. Machine learning models can also learn from historical storage data and improve prediction accuracy over time. In this way, AI can move post-harvest sugar beet management from a reactive approach to a more predictive and preventive system. Such intelligent monitoring can reduce manual inspection, improve decision-making, and support more sustainable storage practices.

Artificial intelligence (AI) technologies have been widely applied to improve post-harvest management and quality assessment in sugar beet systems (Table 5). Deep learning and computer vision approaches enable automated inspection of harvested roots, allowing accurate detection of defects and quality classification using image-based datasets [98]. In addition, semantic segmentation techniques have demonstrated high precision in identifying individual sugar beets and detecting surface irregularities during post-harvest handling, achieving accuracy levels of approximately 98% [109]. Machine vision systems combined with deep learning algorithms further support the detection of mechanical damage and disease symptoms during harvesting and storage processes [110].

Moreover, the integration of AI with environmental sensor data enables continuous monitoring of storage conditions, facilitating early identification of deterioration and improving decision-making [102]. AI-driven data analytics and remote sensing technologies also contribute to optimizing post-harvest logistics, including transportation planning and storage scheduling, thereby reducing quality losses [21]. Overall, these advancements highlight the significant potential of AI-based systems in enhancing efficiency, accuracy, and sustainability in sugar beet post-harvest management.

### 5.4. Role of IoT in Sugar Beet Production

The adoption of IoT technologies in agriculture leads to the generation of extensive volumes of data, which can be leveraged to extract meaningful and actionable insights. Next-generation sugar crop production faces two critical challenges: handling the complexity of multimodal agricultural data and ensuring transparency in artificial intelligence, including interpretability and ethical considerations [111]. AI-driven models must remain robust under diverse and dynamic field conditions while also being interpretable for farmers to build trust and support informed decision-making processes [112]. In addition, the development of autonomous robotic systems should account for varying climatic stresses and operate safely without causing damage to crops during extreme environmental events. Addressing these challenges requires a collaborative effort involving farmers, engineers, policymakers, and researchers to fully realize the potential of agricultural robotics for sustainable sugar crop production [113]. The IoT sensors and systems implemented in sugar beet production can be grouped into environmental sensing, soi sensing, plant health monitoring, phenotyping remote sensing with UAVs, and climate monitoring systems as elaborated in Table 5. Furthermore, the effective adoption of advanced agricultural technologies among smallholder sugar crop farmers demands strategies tailored to their specific limitations and needs. In regions such as sub-Saharan Africa, small-scale producers often encounter restricted access to modern farming infrastructure and advanced agricultural systems. Conventional agricultural storage often uses fixed cooling or ventilation cycles, which can consume a large amount of energy.

### 5.5. Data Privacy and Cybersecurity in Agricultural IoT

Smart farming operations leverage distributed remote sensors to constantly evaluate climate and soil metrics. This data is broadcasted to user-facing applications via SMS and web interfaces, while simultaneously being processed by predictive algorithms to facilitate autonomous mechanical interventions, such as self-activating irrigation networks [114]. Many field sensors have limited security features and may use weak login systems or poorly protected communication channels. This can be risky in smart sugar beet storage because incorrect or manipulated sensor data may hide early signs of spoilage. For example, false VOC or temperature readings could delay the detection of fermentation and lead to serious storage losses [115]. A comprehensive IoT agricultural framework utilizes wireless sensor networks and AI predictive models to track environmental metrics, delivering real-time alerts and enabling automated farm management interventions like smart irrigation [116].

### 5.6. Computational Requirements and Real-Time Feasibility for Industrial Applications

M Advanced sensing technologies are gaining widespread adoption in grassland monitoring and urban agriculture. Internet of Things (IoT)-based automated irrigation and environmental control systems are increasingly utilized in vertical farming and hydroponic production to improve resource efficiency and enhance crop productivity [117]. In addition, when it comes to beet piles dense beet piles, metal structures, long transmission distances, and rural storage locations can weaken wireless signals and make continuous cloud connectivity difficult to maintain.

To overcome these challenges, Edge AI offers a practical solution for industrial deployment. Instead of sending all raw data to the cloud, computation can be performed closer to the sensing location using embedded devices or local microcontrollers. Lightweight machine learning models, such as Random Forests, can be implemented on edge devices to analyze multi-gas sensor data directly at the storage site [118]. In this configuration, only essential information, such as spoilage alerts, anomaly flags, or periodic summary data, is transmitted to a cloud dashboard. This hybrid edge–cloud approach can reduce bandwidth demand, improve response time, support real-time monitoring, and increase the operational lifetime of sensor nodes in industrial sugar beet storage systems [119]. IoT-based systems can make storage management more efficient by activating ventilation only when sensors detect signs of physiological stress, microbial activity, or increased respiration. This targeted control can reduce unnecessary energy use while helping maintain better storage conditions [120]. Table 6 summarizes the various applications of IoT sensors used in sugar beet production.

Preventing even a small amount of post-harvest rot in large industrial storage piles can provide a quick return on investment. Early detection of unusual changes allows storage managers to identify and process deteriorating beets before spoilage spreads to other parts of the pile. This helps protect sucrose yield, reduce economic losses, and maintain market value [121]. As the cost of edge computing microcontrollers and energy harvesting IoT devices continues to decline, AI-based storage monitoring is becoming more affordable and practical. This makes the technology increasingly suitable not only for large industrial cooperatives but also for smaller agricultural businesses [122].

**Table 6:** Applications of IoT Sensors in Sugar Beet Production.

Sensor Category	Application Description	References
Environmental Sensors	Monitor atmospheric conditions such as temperature, humidity, and surrounding climate data	[123]
Soil Sensors	Measure key soil properties including moisture content, salinity, and pH levels	[124]
Plant Health Sensors	Evaluate plant physiological conditions and detect early signs of stress or disease	[125]
Smart Imaging Sensors	Enable large-scale crop monitoring using UAVs or satellites with advanced imaging methods	[126]
Climate Monitoring Sensors	Track environmental variables such as rainfall, temperature, and humidity over time	[124]

## 6. Challenges and Future Directions in Sugar Beet Storage Monitoring

Sugar beet storage monitoring still faces several challenges that reduce the effectiveness of current sensing and data-based systems. One major issue is the harsh and uneven conditions inside beet piles. Factors such as temperature changes, high moisture, and poor airflow create a complex environment that affects sensor accuracy and stability. These conditions also influence how gases behave, making it difficult to consistently detect and measure volatile compounds during storage.

From a technical point of view, using wireless sensor networks inside dense beet piles is also challenging. Signals can weaken or get blocked, especially when using low-power communication systems, which may lead to missing or delayed data. In addition, choosing the right sensors is not straightforward. Different sensors respond differently to gases like ethanol and other compounds related to microbial activity, which can affect the reliability of the measurements.

Another important limitation is the lack of fully integrated systems that can measure multiple parameters at the same time, such as gases, temperature, and humidity. Many current systems focus on only one type of data, which makes it harder to fully understand how decomposition develops over time. Also, most sensor calibration is done in controlled lab settings, which does not represent real storage conditions, leading to inaccurate results in practice.

Environmental changes further increase the difficulty of monitoring. Variations in temperature, humidity, and biological activity can cause inconsistent sensor readings. In addition, long-term issues such as sensor drift, reduced sensitivity, and maintenance problems in humid conditions make continuous monitoring more difficult. To overcome these challenges, future work should focus on developing integrated monitoring systems that combine multiple sensors with reliable wireless communication. Improving sensor calibration under real storage conditions is essential. The use of machine learning can help analyze complex data and detect early signs of spoilage caused by microbial activity. Furthermore, designing energy-efficient sensor networks and improving communication methods will be important for large-scale deployment. The use of edge computing and cloud platforms can support real-time data analysis and decision-making. By addressing these challenges, more reliable monitoring systems can be developed to reduce storage losses and improve the overall management of sugar beet storage.

**Figure 7:**
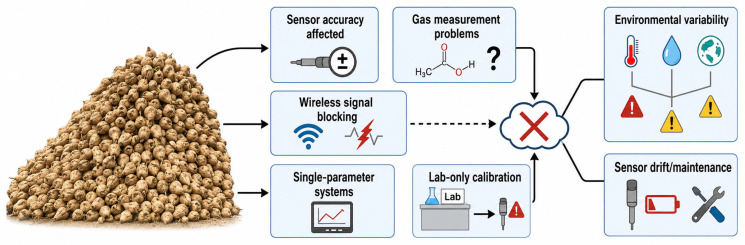
Challenges in sugar beet storage monitoring, including sensor limitations, wireless signal blocking, environmental variability, and calibration issues. Solid arrows show direct physical impacts, dashed arrows indicate interrupted data flow, and the “X” denotes cloud connectivity failure.

Figure 7 shows the major challenges associated with sugar beet storage monitoring systems as shown the sugar beets are stored in a bucket to replicate the storage conditions of the sugar beets. It shows how harsh storage conditions, such as high humidity, temperature variation, and limited airflow, can negatively affect sensor accuracy and stability. The figure also highlights difficulties in gas measurement, particularly for compounds related to microbial activity during decomposition. In addition, wireless communication issues, such as signal blocking within dense beet piles, can lead to unreliable data transmission. The use of single-parameter sensing systems and lab-based calibration further limits the ability to capture real storage conditions accurately. Environmental variability and long-term sensor drift also reduce system reliability over time. Overall, the figure emphasizes the need for more robust, integrated, and adaptive monitoring solutions for effective sugar beet storage management. VOC-based monitoring is highly relevant for sugar beet storage in the northern United States, particularly in Minnesota, North Dakota, and Montana, where harvested roots are often stored under cold seasonal conditions before processing. Minnesota was projected to produce nearly 12.0 million tons of sugar beet in 2024, and Montana production is mainly located in the Yellowstone River growing region [127,128]. During pile storage, maintaining suitable cold or frozen conditions is essential because thawing, restricted airflow, moisture buildup, and localized microbial growth can accelerate root deterioration and reduce sugar recovery [129,130]. Therefore, VOC monitoring systems designed for ND, MN, and MT storage environments should consider pile-scale variability, winter temperature shifts, ventilation differences, and wireless signal attenuation within dense beet piles.

### Data Quality, Sensor Calibration, and Sensor Drift in Long-Term Storage

The effectiveness of AI-based decision-making in post-harvest management largely depends on the accuracy and reliability of sensor data. During long-term sugar beet storage, ensuring high-quality data can be challenging because storage piles often experience harsh conditions such as high humidity, condensation, significant temperature change, and the accumulation of dirt, all of which can affect sensor performance [131]. These factors may introduce noise into the collected data, making it more difficult to detect the early signs of microbial activity. As a result, ML models used in sensor data analytics may struggle to differentiate between actual spoilage processes and temporary changes caused by environmental conditions. In addition, sensor measurements must be carefully calibrated under practical operating conditions of sensors to ensure that the collected information can be reliably used for decision-making. Calibration procedures should represent actual field and storage environments rather than relying solely on controlled laboratory settings. Moreover, the calibration range of the sensor readings should encompass the whole possible case scenarios of sensing in real conditions, which is itself is a challenging issue. In addition, reliable interpretation of raw sensor data depends on regular baseline adjustments and data-processing methods that account for the fluctuating temperature and humidity conditions present within sugar beet storage piles [132]. Signal drift is one of the major limitations in long-term monitoring applications. During storage periods that may extend over several months, the sensitive layers of chemical and gas sensors can gradually deteriorate due to prolonged exposure to volatile compounds, moisture, and other contaminants. This deterioration leads to a slow shift in the baseline response of the sensors, which may result in inaccurate readings by showing artificially increased or decreased gas concentrations and volatile organic compounds over storage time of sugar beet piles [133].

## 7. Conclusions

The integration of artificial intelligence, IoT, and VOC sensing provides a strong opportunity to reduce post-harvest sucrose losses in the sugar beet industry. Recent developments in electronic nose systems, gas sensors, and machine learning models have improved the early detection of storage deterioration. However, practical use in commercial storage facilities is still limited by uneven conditions within large beet piles, sensor drift, wireless signal loss, and delays caused by cloud-based data processing. To address these challenges, future systems should move toward edge computing architectures, where key spoilage indicators such as ethanol and ethyl acetate can be analyzed directly at the sensor node.

The next stage of post-harvest sugar beet monitoring should move beyond isolated sensor units toward integrated AI–IoT–VOC monitoring platforms. Instead of treating gas detection, environmental sensing, and data analysis as separate functions, future systems should combine multiple data sources through sensor fusion. By analyzing VOC emissions together with temperature changes, humidity distribution, CO_2_ levels, and spatial storage conditions, AI models can better confirm abnormal patterns, reduce false spoilage alerts, and improve diagnostic accuracy [134].

Another promising direction is the use of digital twin technology for sugar beet storage management. In this approach, real-time IoT and VOC data can be used to update a virtual model of the physical storage pile. Such a model could help facility managers simulate how heat, moisture, and spoilage-related gases move through dense beet piles and predict where localized deterioration may develop [135]. This would support earlier intervention and more targeted storage control.

To support the transition from research to commercial deployment, standardized VOC datasets for post-harvest sugar beet storage are also needed. These datasets should connect VOC profiles with confirmed microbial activity, storage temperature, humidity, pile location, and beet quality changes. This would allow AI models to be trained, compared, and validated across different storage conditions and geographic regions. In addition, future hardware development should focus on low-cost, durable, biodegradable, and energy harvesting IoT sensor nodes. Such sensors could reduce maintenance requirements and may allow safe use in large industrial beet piles without the need for labor-intensive retrieval after storage [136].

For industrial implementation, AI-based VOC monitoring systems should be modular and compatible with existing control infrastructure, including Programmable Logic Controllers (PLCs) and Supervisory Control and Data Acquisition (SCADA) systems. Instead of functioning only as separate monitoring dashboards, these systems should be able to support automatic decisions, such as activating ventilation or cooling when early fermentation indicators are detected. This type of closed-loop monitoring could improve energy efficiency, reduce spoilage risk, protect sucrose yield, and make AI- and IoT-supported sugar beet storage more practical for commercial use.

## Figures and Tables

**Table 1:** Comparison of AI, IoT, and VOC sensing approaches for sugar beet monitoring.

Technology	Sugar Beet Application	Key Advantage	References
Deep Learning	Weed and disease detection	High visual precision	[41]
Machine Learning	VOC classification and shelf-life prediction	Fast; edge-friendly	[42]
Cloud IoT	Remote storage climate monitoring	Supports cloud analytics	[43]
Edge IoT	Real-time spoilage alerts	Low latency; works offline	[44]
MOS Arrays	Ethanol and ester sensing	Low-cost; scalable	[45]
GC–MS	Metabolic gas validation	High chemical accuracy	[46]

**Table 2:** Volatile Organic Compound and their description based on boiling point [66].

Volatile Organic Compounds	Boiling Point in (°C)	Description
Very volatile organic compounds (VVOCs)	<0 to 50–100	Lowest boiling points, highly volatile.
Volatile organic compounds (VOCs)	50–100 to 240–260	Moderate boiling points, evaporate easily.
Semi-volatile organic compounds (SVOCs)	240–260 to 380–400	High boiling points, evaporate slowly.

**Table 3:** Types of sensors used to detect volatile organic compounds and their advantages.

Sensor Type	Typical Range/LOD	Sensitivity Indicator	Selectivity	Ref.
MOS	1–10,000 ppm; LOD: ~0.1–10 ppm	High response; temperature dependent	Low–moderate; humidity/cross-gas interference	[82]
MOSFET	1–1000 ppm; LOD: ~0.1–5 ppm	Fast low-ppm electrical response	Moderate–high with catalytic gate materials	[83]
Piezoelectric	10–10,000 ppm; LOD: ~1–10 ppm	Mass-loading response at room temperature	Moderate; coating dependent	[84]
QCM	0.1–1000 ppm; LOD: ~0.01–1 ppm	High frequency shift sensitivity	Moderate–high with selective coatings	[85]
SAW	0.01–1000 ppm; LOD: ~0.001–0.1 ppm	Very high surface-mass sensitivity	Moderate–high with functional coatings	[87]
Electrochemical	0.1–500 ppm; LOD: ~0.01–1 ppm	Linear low-power gas response	Moderate–high for target gases	[86]
Optical/PID/NDIR	0.001–10,000 ppm; LOD: ppb–ppm	High optical/ionization sensitivity	PID broad VOC; NDIR gas-specific	[95]
MS-based	0.0001–10,000+ ppm; LOD: ppt–ppb	Ultra-high analytical sensitivity	Very high compound identification	[96]

**Table 4:** AI Applications in sugar beet production.

AI	Application	Key Findings	Ref.
CNN/Deep Learning	Disease detection	Deep learning models enable accurate detection of sugar beet leaf diseases using image analysis	[98]
Hyperspectral Imaging + ML	Early disease detection	Machine learning models combined with hyperspectral imaging achieve high accuracy in identifying infected plants	[99]
UAV + Computer Vision	Crop monitoring	Aerial imagery and AI models can detect plant health conditions and crop stress across large fields	[100]
ANN/ML Models	Yield prediction	Machine learning techniques provide reliable predictions of root yield and sugar content	[101]
Deep Learning + Remote Sensing	Plant detection	AI-based remote sensing enables automated monitoring and mapping of sugar beet fields	[102]

**Table 5:** Applications of artificial intelligence in post-harvest management of sugar beet.

AI Technology	Application	Key Findings	Ref.
Deep Learning (CNN) + Computer Vision	Post-harvest quality inspection	Automated detection of harvested sugar beet roots, damage identification, and quality classification using RGB image datasets	[98]
Semantic Segmentation + AI	Beet detection and defect identification	AI models achieved high detection accuracy (~98%) for identifying individual sugar beets and surface defects during post-harvest handling	[109]
Machine Vision + Deep Learning	Mechanical damage detection	AI-based vision systems can detect damaged or diseased sugar beet roots during harvesting and storage operations	[110]
AI + Sensor Monitoring	Storage condition monitoring	AI models combined with environmental sensors enable monitoring of storage conditions and early detection of deterioration	[102]
AI + Remote Sensing/Data Analytics	Post-harvest logistics optimization	AI-driven analytics support better planning of beet transport, storage scheduling, and processing timing to reduce quality losses	[21]

## Data Availability

No new data were created or analyzed in this study. Data sharing is not applicable to this article.
